# Investigating the cost-effectiveness of videotelephone based support for newly diagnosed paediatric oncology patients and their families: design of a randomised controlled trial

**DOI:** 10.1186/1472-6963-7-38

**Published:** 2007-03-05

**Authors:** Mark Bensink, Richard Wootton, Helen Irving, Andrew Hallahan, Deborah Theodoros, Trevor Russell, Paul Scuffham, Adrian G Barnett

**Affiliations:** 1 The University of Queensland Centre for Online Health, Brisbane, Australia; 2 Haematology, Oncology and Stem Cell Transplant Unit, Royal Children's Hospital, Brisbane, Australia; 3 Griffith University School of Medicine, Logan, Australia; 4 The University of Queensland School of Population Health, Brisbane, Australia

## Abstract

**Background:**

Providing ongoing family centred support is an integral part of childhood cancer care. For families living in regional and remote areas, opportunities to receive specialist support are limited by the availability of health care professionals and accessibility, which is often reduced due to distance, time, cost and transport. The primary aim of this work is to investigate the cost-effectiveness of videotelephony to support regional and remote families returning home for the first time with a child newly diagnosed with cancer

**Methods/design:**

We will recruit 162 paediatric oncology patients and their families to a single centre randomised controlled trial. Patients from regional and remote areas, classified by Accessibility/Remoteness Index of Australia (ARIA+) greater than 0.2, will be randomised to a videotelephone support intervention or a usual support control group. Metropolitan families (ARIA+ ≤ 0.2) will be recruited as an additional usual support control group. Families allocated to the videotelephone support intervention will have access to usual support plus education, communication, counselling and monitoring with specialist multidisciplinary team members via a videotelephone service for a 12-week period following first discharge home. Families in the usual support control group will receive standard care i.e., specialist multidisciplinary team members provide support either face-to-face during inpatient stays, outpatient clinic visits or home visits, or via telephone for families who live far away from the hospital. The primary outcome measure is parental health related quality of life as measured using the Medical Outcome Survey (MOS) Short Form SF-12 measured at baseline, 4 weeks, 8 weeks and 12 weeks. The secondary outcome measures are: parental informational and emotional support; parental perceived stress, parent reported patient quality of life and parent reported sibling quality of life, parental satisfaction with care, cost of providing improved support, health care utilisation and financial burden for families.

**Discussion:**

This investigation will establish the feasibility, acceptability and cost-effectiveness of using videotelephony to improve the clinical and psychosocial support provided to regional and remote paediatric oncology patients and their families.

## Background

The Royal Children's Hospital (RCH) Haematology, Oncology and Stem Cell Transplant Unit is the major tertiary paediatric referral centre for Queensland, northern New South Wales and the southwest Pacific. A multidisciplinary team of medical, nursing and allied health professionals provides care and support to children with cancer, as well as their families. Each year the service cares for around 100 newly diagnosed patients.

Childhood cancer presents a major life stressor for the entire family [1,2]. Significant changes to the everyday lives of families, practically, socially and emotionally, cause major disruption [3-5]. There is evidence that this disruption results in isolation and poor communication between family members, anxiety, low self-esteem and school problems for siblings [6] and anxiety, post-traumatic stress symptoms and risk of depression for parents [7,8]. Families supporting home care of their child are faced with personal and financial sacrifice, higher risk of fatigue and burnout, and the prospect of managing significant symptomatology in their child. Their own mental and emotional health is directly affected [9] as they experience high levels of depression and anxiety. Providing ongoing support to these families is an essential part of care [10-13].

**Figure 1:**
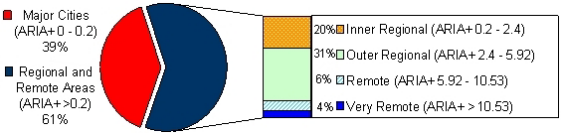
Proportion of newly diagnosed paediatric oncology patients in major cities and regional and remote areas (data provided by the Royal Children's Hospital January 2004 to January 2006, n = 180).

Around 60% of the families cared for by the RCH (Figure 1) live in regional and remote areas (based on post code and corresponding ARIA+ score – Accessibility/Remoteness Index of Australia [14,15]. For families living in regional and remote areas, the opportunities to receive specialist support are inhibited by a number of factors [16] These include: availability of health care professionals [17] and accessibility due to distance, time, cost and transport [18]. One possible solution is the use of online support mechanisms, such as videotelephony, to help give much needed support to patients, parents, siblings and the family as a whole.

Studies investigating the use of videotelephony to date have been mostly small scale projects focusing on feasibility. The potential of videotelephony in adult home nursing has been shown in pilot trials in the UK [19], the US [20] and in Australia [21]. Low-quality compressed video can be used to support care directly to the home. One small study (n = 20) identified that whilst there are activities that cannot be conducted by videotelephone, those that can coincide with the typical interactions occurring in face-to-face home visits [22].

A larger scale exploratory investigation into the use of videotelephones for psychotherapy (n = 80) reported higher scores for client levels of initiative, trust and spontaneity via videotelephone than the same scores obtained for face-to-face therapy [23]. Although this study did not deliver services directly to patient homes (instead patients were assessed via videotelephone between separate rooms in the treating facility) it does provide evidence that videotelephony can be used to provide the type of personal psychosocial support required by paediatric oncology patients and families. The most extensive study using videotelephones to support adult home care (n = 212) [24] showed that:

• clinical and educational services can be delivered successfully using videotelephones and the standard home telephone network

• the quality of care delivered via virtual videotelephone home visits was comparable to face-to-face home visits with the added benefit of cost savings.

The only other evidence in the literature is in the area of adult oncology. Videotelephones have been used successfully since 1998 to provide direct patient care and supportive care [25], as well as supporting palliative care delivery for patients in their home [26,27]. This has been shown to be a cost-effective service delivery method [28]. A large randomised controlled trial in adult palliative care is also underway in Canada [29]. To date there have been no published studies investigating the use of videotelephony for supporting paediatric oncology families.

### Hypotheses

1. Videotelephony will be a feasible and acceptable mode of service delivery to regional and remote paediatric oncology families

2. Support provided using videotelephony to regional and remote paediatric oncology families will be as effective as support provided via traditional mechanisms to metropolitan families

3. Using videotelephony will be a cost-effective method of service delivery.

## Methods/design

### Design

This study is a randomised controlled trial using three-groups: (1) Regional and remote intervention group, (2) Regional and remote control group, and (3) Metropolitan control group. Families will be classified as either metropolitan or regional and remote based on the ARIA+ score using the post code of the family's primary place of residence. Regional and remote families will be randomly allocated either to the intervention (1), or to a usual support control group (2). Metropolitan patients will receive support via traditional means as an additional usual support control group (3). This group will be used to provide a comparator against which the support provided to the regional and remote families can be judged.

The protocol for this study is registered with the Australian Clinical Trials Registry (ACTRN 012606000487516) and has been approved by the Royal Children's Hospital and Health Service District Ethics Committee and the University of Queensland Behavioural and Social Sciences Ethical Review Committee.

### Participants

Patients of the Royal Children's Hospital Haematology, Oncology and Stem Cell Transplant Unit will be identified by their paediatric oncologist before first discharge home. To be included in the study patients will be: newly diagnosed male or female paediatric oncology patients aged 2–18 years. Patients will be excluded if a home telephone connection of satisfactory quality cannot be installed.

The paediatric oncology clinical nurse consultants responsible for each patient will approach the family individually. Families will be asked to participate in a project investigating the use of videotelephones for supporting children with cancer and their families. A brief overview of the project will be provided before each family is asked if they consent to talk with a research assistant about the project.

The research assistant responsible for the project will provide written information on the trial informing all potential participants about the study, the frequency, content and expected duration of telephone interviews and what their participation would entail before obtaining consent.

### Randomization

An independent researcher with no direct involvement in the trial will be responsible for random allocation. A table of random numbers will be generated. Even numbers will denote allocation of regional and remote participants to the control group and odd numbers to the intervention group. Allocations to either group based on the table of random numbers will be written on a piece of paper and sealed in sequentially numbered opaque envelopes. As regional and remote families are enrolled in the trial, the research assistant will obtain information on allocation from the envelopes in sequential order and assign participants to their designated group. Due to the interactive nature of the intervention it is not possible to blind clinicians providing the intervention or participants involved in the study.

### Interventions

#### Usual care

All patients regardless of location are conventionally supported at first discharge home by the specialist multidisciplinary team of medical, nursing and allied health professionals. This is either done face-to-face during outpatient clinic visits or during subsequent inpatient stays, or via telephone to families who live far from the hospital. Telephone calls between specialist team members and families at home are used to assist with: problems that arise, clinical assessment and management and to provide education and counselling. Patients and families in the usual support control groups (i.e., all metropolitan families and those regional and remote families randomly allocated to the regional and remote control group) will have access to all of these standard services and facilities.

#### Videotelephone support

Patients randomly allocated to the regional and remote intervention group will have access to all of the usual support plus the videotelephone service. The videotelephone service can be used for: education, communication, counselling and monitoring with specialist multidisciplinary team members. The videotelephone service will be available to families for a 12-week period following first discharge home (families will be able to request a videotelephone call with team members at any time, the oncology clinical nurse consultant will call the family home twice a week for the first four weeks and weekly thereafter using the standard telephone and will initiate subsequent videotelephone calls with team members as required). In addition, the videotelephone will be available to family members to facilitate intra-family support during inpatient stays.

### Measurement

The SF-12v2 will be used to measure the primary outcome measure, parental health related quality of life. The SF-12 is a valid and reliable measure [30,31] used in a number of countries including Australia. More recently the SF-12 has been used in caregiver quality of life research [32].

Secondary outcome measures for parents will be: the informational/emotional subscale of the Medical Outcomes Study (MOS) Social Support Survey (a valid and reliable instrument for quantifying social support [33]), the 4-item Perceived Stress Scale (PSS) [34] and the FAMCARE scale (a valid and reliable instrument designed specifically to assess satisfaction with advanced cancer care [35]). For patients and siblings the Pediatric Quality of Life Inventory (Peds QL) parent proxy will be used (a valid and reliable measure of quality of life in children applicable to healthy populations and those with chronic disease [36]). In addition the Health Utilities Index Mark 2 (HUI2) will be completed by parents and used to assess patient quality-adjusted life years (QALYs).

Data collection will be undertaken via: user logs, chart audits, service database reviews, telephone and in-person interviews. Data collection with parents will begin at baseline (after consent has been obtained). The interviewer will be blinded to group allocation. Interviews will be used to obtain: demographics, baseline SF-12 scores, baseline informational and emotional support scores, baseline patient and sibling quality of life scores, and baseline satisfaction scores.

To reduce participant burden the completion of the various interview components will be staggered (Table 1). The initial interview will be the longest.

**Table 1:** Interview schedule.

Component	Week												
	0	1	2	3	4	5	6	7	8	9	10	11	12
Demographics													
SF-12v2													
Informational/emotional support													
Perceived stress													
Patient quality of life													
Sibling quality of life													
HUI2													
Satisfaction													
Health care utilisation													
Family costs													

### Data analysis

#### Sample size calculations

A total of 162 participants will be included in the study (based on data provided by the tertiary paediatric oncology referral centre this is feasible for a 24-month trial with a 20% refusal/attrition rate), 108 in the control group – 54 metropolitan and 54 regional and remote, and 54 in the regional and remote intervention group. Sample size calculations are given for the two main comparisons and economic analyses:

1. Regional and remote intervention group vs regional and remote usual support control group

- To test whether the provision of videotelephones to families in regional and remote areas can improve the health related quality of life of parents caring for a child with cancer over usual care, a unpaired t-test of the mean difference will be used [38]. This will be based on the SF-12 at week 12.

- As no paediatric caregiver research using the SF-12v2 has been undertaken in Australia to date, published evidenced available on paediatric parental caregiver burden in the US [39] and US norms [31] (Table 2) have been used as a guide for the sample size calculation. A clinically important difference would be to improve mean mental health related quality of life of parents (SF-12 Mental Component Summary [MCS]) in the intervention group by 7.5 points. This figure is further supported by calculation of SF-12v2 standard error of measurement [SEM], calculated as the standard deviation of an instrument multiplied by the square root of one minus its reliability coefficient [40]. With an SD of 10 and reliability coefficient of 0.86 [31], the MCS-12 has an SEM of 3.74. Evidence by Wywrich [41] supports the use of one SEM as an approximation of the *minimal *clinically important difference [MCID] of a psychometric instrument. Using this approximation, an improvement in MCS-12 of 7.5 represents two times the MCID, which is a large clinically important difference.

**Table 2:** Published evidence available on the SF-12 health-related quality of life instrument.

Context	n	Details	Mean	SD
The impact of paediatric tracheotomy on parental caregiver burden and health status (US)[39]	154	PCS-12 MCS-12	50.5 35.8	11.3 11.4
US Norms[31]	7069	General US population		
		PCS-12	49.63	9.91
		MCS-12	49.37	9.75
		Depression		
		PCS-12	45.55	11.71
		MCS-12	37.40	10.76

- Based on an improvement in MCS-12 mean in the intervention group of 7.5, and using SF-12v2 norm-based scoring methods (US general population SF-12v2 mean of 50 and SD of 10 [31]), at a power of 90% and a two-sided significance level of 1%, a sample size of 108 will be required, i.e. 54 in the intervention group and 54 in the control group.

2. Regional and remote intervention group vs metropolitan usual support control group

- To test whether the provision of videotelephones to families in regional and remote areas can improve the mental health related quality of life of parents to a level equivalent to that of metropolitan families receiving usual support, a test of equivalence will be used (according to the equivalence method described by Matthews [42]).

- With a two-sided significance level of 1%, sample size of 54, a mean difference between groups of zero and an equivalence bound of plus/minus 7.5, the study has a power of 87.2% for finding a genuine equivalence.

#### Statistical analysis

Data analysis will be on an intention-to-treat basis and consist of univariate analysis between randomised groups on the primary and secondary outcomes. A more in-depth analysis will use longitudinal methods to account for correlations within families and to examine changes over time.

#### Economic analysis

It is hypothesised that using videotelephony will be a cost-effective method of service delivery. Economic evaluation will be investigated in four areas.

1. The cost of providing improved support

- The potential for videotelephony to improve the health related quality of life of parents comes at a cost. The total cost of providing the videotelephone service will be calculated including project establishment costs, equipment costs, maintenance costs, communication costs and staff costs.

2. Health care use savings

a. *Use of local health services*

- The improved support provided by videotelephony has the potential to reduce the need for parents and patients to access local health care services. These include (but are not limited to) community nursing visits, general practitioner consultations, psychosocial health care consultations, respite care, emergency room visits and local hospital admissions. The improved support is not intended to minimise the much needed and valued local community support. Instead, the improved support provided by the videotelephone service reduces the need to burden these services for reasons that can be avoided with improved specialist support (i.e. improved symptom management, improved continuity of care, improved psychosocial support, improved care planning and improved emergency management).

b. *Use of the entire health system*

- To investigate the effect on costs to the health system, health care use data will be gathered. Information on health care encounters (including in-patient, outpatient, emergency department, general practitioner, home care and respite care instances) will be collected from families in the intervention and control groups with bi-weekly telephone interviews. Hand searching of patient medical records will be completed to determine and verify all health care encounters. Encounters will be valued according to the Australian Medicare Benefits Schedule of resource items and their associated costs.

3. Reduced financial burden for families

- Families may also benefit financially from the improved support provided by the videotelephone service. Improved support has the potential to reduce the need for travel and associated costs. Information on family out-of-pocket expenses (such as travel costs including fuel, parking, fares; and other costs including meals, child care and accommodation) will also be obtained to assess the direct non-medical financial impact on families and how effectively the videotelephone service reduces this impact.

4. Cost-effectiveness analysis

- The health system perspective will be used for the primary analysis, and will be augmented in subsequent analysis with direct non-medical costs. The costs of providing the videotelephone service plus the total cost of health service use for the intervention group will be compared to the total cost of health service use identified in the regional and remote usual support control group. This is the incremental cost.

- Using the *Research Instruments *listed above, differences between the intervention group and the regional and remote usual support control group will be calculated. From this, the incremental cost per additional unit of benefit gained will be calculated, i.e. the incremental cost-effectiveness ratio (ICER); e.g. it costs an additional $x to improve parental quality of life by 1 point.

- A cost-utility analysis will also be undertaken. This allows comparisons between programs to be made and hence value for money judgements, as outcomes are standardised into quality-adjusted life years (QALYs). QALYs will be calculated using the HUI2 and the SF-6D algorithm to convert the SF-12 scores into utility scores (where 0 = dead, and 1.0 = best imaginable health state) and multiplied by the duration of life in that health state [37]. Differences in QALYs (for both parents and patients) between groups will be used to estimate the incremental cost-utility ratios.

- Sensitivity analyses will be conducted around the variables with the greatest uncertainty as well as the duration of final utility score at the end of the 12-week follow-up.

## Discussion

In 2001, over 600 children were diagnosed with cancer in Australia [43]. By 2011, the incidence of childhood cancer is projected to increase by 7% for females and 5% for males [44]. The cumulative toll on these children and their families is high, socially, psychologically and economically. They have a greater risk of developing psychological and emotional difficulties than other children and families [45]. The support provided by specialist health professionals, the continuity of follow-up care and the support provided by family members have been identified as critical factors [46].

Improving the specialist support provided to these families has the potential to increase the quality of life of the entire family through: improved symptom identification and management, the provision of information, education and counselling, and better continuity of care. This may also reduce the need for these families to access local health care services for reasons that can be avoided with improved specialist support. It also has the potential to reduce some of the economic burden these families face through reductions in unnecessary travel.

The use of videotelephony will overcome many of the issues associated with providing improved support at a distance including the availability of experienced specialist health care professionals in regional and remote areas, and accessibility difficulties due to distance, time, cost and transport.

This work will present a new and innovative use of videotelephone technology as well as a new method for supporting regional and remote oncology patients and their families. The completion of a large randomised controlled trial will add significantly to the small evidence base for home telehealth [47]. It will be the first study of its kind in paediatric oncology. With the inclusion of a comprehensive economic evaluation, it will set an international benchmark as very few studies in home telehealth include cost-effectiveness or cost utility analyses [48]. It will also assist with the application of this model of support in other health care contexts.

## Competing interests

The author(s) declare that they have no competing interests.

## Authors' contributions

All authors have contributed to the development of the study and procurement of funding. MB conceived of the project in consultation with RW, HI and AH. DT and TR have worked with MB and RW in the development of the videotelephone technology and its application to paediatric oncology health service provision. PS has worked with MB and RW to design the economic evaluation methodology. AB has worked with MB and RW to provided assistance with the sample size calculation and statistical analyses for the study. MB drafted the manuscript with input from all other authors who have read and approved of the final version.

## Pre-publication history

The pre-publication history for this paper can be accessed here:

http://www.biomedcentral.com/1472-6963/7/38/prepub
